# Ecological and Societal Services of Aquatic Diptera

**DOI:** 10.3390/insects10030070

**Published:** 2019-03-14

**Authors:** Peter H. Adler, Gregory W. Courtney

**Affiliations:** 1 Department of Plant and Environmental Sciences, Clemson University, 130 McGinty Court, E143 Poole Agricultural Center, Clemson, SC 29634-0310, USA; 2 Department of Entomology, Iowa State University, 2310 Pammel Drive, 401 Science II, Ames, IA 50011-3222, USA; gwcourt@iastate.edu

**Keywords:** bioassessment, bioinspiration, biological control, climate change, ecosystem engineer, extinction, extremophiles, forensics, keystone species, symbiosis

## Abstract

More than any other group of macro-organisms, true flies (Diptera) dominate the freshwater environment. Nearly one-third of all flies—roughly 46,000 species—have some developmental connection with an aquatic environment. Their abundance, ubiquity, and diversity of adaptations to the aquatic environment position them as major drivers of ecosystem processes and as sources of products and bioinspiration for the benefit of human society. Larval flies are well represented as ecosystem engineers and keystone species that alter the abiotic and biotic environments through activities such as burrowing, grazing, suspension feeding, and predation. The enormous populations sometimes achieved by aquatic flies can provide the sole or major dietary component for other organisms. Harnessing the services of aquatic Diptera for human benefit depends on the ingenuity of the scientific community. Aquatic flies have played a role as indicators of water quality from the earliest years of bioassessment. They serve as indicators of historical and future ecological and climate change. As predators and herbivores, they can serve as biological control agents. The association of flies with animal carcasses in aquatic environments provides an additional set of tools for forensic science. The extremophilic attributes of numerous species of Diptera offer solutions for human adaptation to harsh terrestrial and extraterrestrial environments. The potential pharmaceutical and industrial applications of the symbiotic microbial community in extremophilic Diptera are better explored than are those of dipteran chemistry. Many flies provide valuable ecological and human services as aquatic immatures, but are also pests and vectors of disease agents as terrestrial adults. The scientific community, thus, is challenged with balancing the benefits and costs of aquatic Diptera, while maintaining sustainable populations as more species face extinction.

## 1. Introduction

From micro-thin trickles to torrential waterfalls, tree holes to open oceans, and glacial meltwaters to hot springs, the ubiquitous Diptera have conquered the aquatic environment. They have been found at elevations up to 5600 m in the Himalayas and at depths of more than 1000 m in Lake Baikal [1]. In addition, they are the only free-living insects to have colonized the aquatic environment on all continents—two species of chironomids inhabit Antarctica [2].

More than any other order of insects, Diptera test the limits of the definition of “aquatic”. Delimiting aquatic Diptera, apart from semi-aquatic Diptera, is largely futile. The vast majority of larval dipterans inhabit wet or damp microhabitats, presenting a nearly seamless series of water-dependent taxa, from those entirely immersed in free water to those in damp substrates or endoparasitic in other organisms. The full range of aquatic to semi-aquatic species can be found in groups such as the Chironomidae and Tipuloidea, and some members are actually terrestrial, although both taxa have conventionally been treated as collectively aquatic [1,3,4,5]. Almost no dipteran passes its entire life cycle in water; minimally, the adult stage is at least temporarily terrestrial. The unique larval habitat—crude petroleum pools and seeps—of the ephydrid *Helaeomyia petrolei* also complicates the definition of an aquatic insect, although the species has typically been considered aquatic [6]. Notwithstanding the challenge of clearly defining aquatic Diptera, we characterize them here as free-living insects that require a wet environment in at least one life stage. More restrictive treatments consider aquatic Diptera to be only those associated with bodies of water [7].

Forty-one (26%) of the 158 families of Diptera have aquatic representatives. These 41 families are scattered throughout the dipteran phylogeny and include nearly 46,000 aquatic species worldwide (Table 1), representing about 30% of all formally described species of Diptera. Thus, Diptera have more aquatic representatives than any other order of insects—at least three times more than the Coleoptera and Trichoptera. All constituent species of about 20 dipteran families are aquatic in at least the larval stage.

We present a synopsis of some of the services provided by aquatic Diptera. These services can be grouped into those of ecosystem importance and those of human importance. The two groupings are not mutually exclusive. Although the aquatic immatures and the terrestrial adults typically provide separate and distinctly different ecological services, we focus on the services provided by the aquatic stages. For context, we begin with a brief overview of the dipteran life cycle and bionomics.

## 2. Life Cycle of Aquatic Diptera

**Figure 1:**
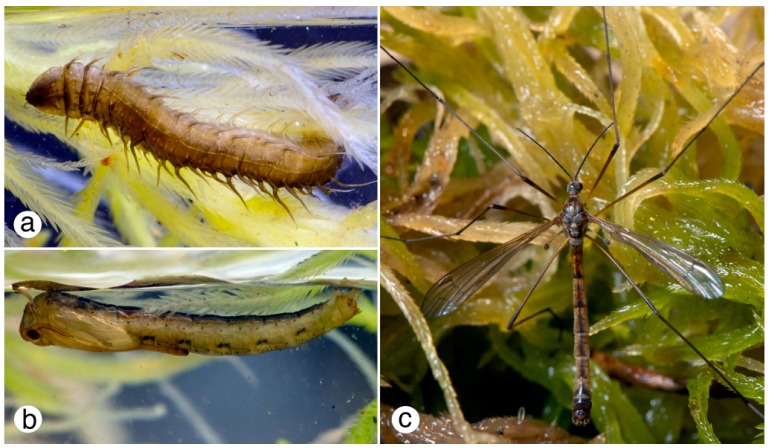
Diptera life cycle. *Phalacrocera tipulina* (Tipuloidea: Cylindrotomidae): (**a**) Final stage (4th instar) larva; (**b**) Pupa; (**c**) Adult male. All figures © G.W. Courtney.

Development in Diptera is holometabolous. A typical life-cycle comprises a brief egg stage (usually of a few days or weeks, but sometimes much longer), at least three larval instars (usually three in Brachycera and four in lower Diptera, but more in Simuliidae, Thaumaleidae, and Tabanidae), a pupal stage of varying length, and an adult stage (imago) that can last from less than two hours (Deuterophlebiidae) to several weeks or even months. In most aquatic taxa, the last-instar larva, pupa, and adult (Figure 1) are the most visible and frequently encountered.

The eggs of aquatic Diptera are usually laid singly, in small clusters, or in loose or compact masses attached to substrates in or near the water. In some groups (e.g., Deuterophlebiidae, Nymphomyiidae, and some Blephariceridae), the female crawls beneath the water to select oviposition sites in or near the larval habitat, which ensures that eggs are placed in a location suitable for larval development. For a given taxon, all larval instars usually occur in the same habitat. In general, the duration of the first instar is shortest, whereas that of the last instar is much greater, often several weeks or months.

The pupae of aquatic Diptera occur in a variety of habitats and generally fall into one of the following categories: (1) free-swimming pupae that rise to the surface at adult emergence (e.g., Chaoboridae, Culicidae, and most Chironomidae); (2) pupae that are attached to benthic substrates and either fully exposed (e.g., Blephariceridae, Deuterophlebiidae, and some Psychodidae) or in a silken cocoon (e.g., Simuliidae and some Chironomidae and Tipuloidea), with the adults emerging from below the water surface; (3) pupae that burrow into marginal substrates after moving, as larvae, to the shoreline (e.g., Athericidae, Tabanidae, and some Tipuloidea); and (4) pupariation, which is a special form of pupation within the integument of the last larval instar (e.g., Ephydridae, Sciomyzidae, Stratiomyidae, and Syrphidae).

Although adults of most aquatic Diptera occur around or near water, species that require a blood meal for ovarian development (e.g., Ceratopogonidae, Culicidae, Simuliidae, and Tabanidae) can occur kilometers from the nearest water source. Some adults spend much of their life skimming over water (e.g., some Chironomidae, Empididae, and Dolichopodidae); however, most groups spend little time directly associated with water. Species that oviposit directly in the larval habitat (e.g., Deuterophlebiidae) enter the water, whereas others merely tap the water gently during oviposition. Although most Diptera exhibit sexual reproduction, parthenogenesis occurs in some groups.

## 3. Bionomics of Aquatic Diptera

**Figure 2:**
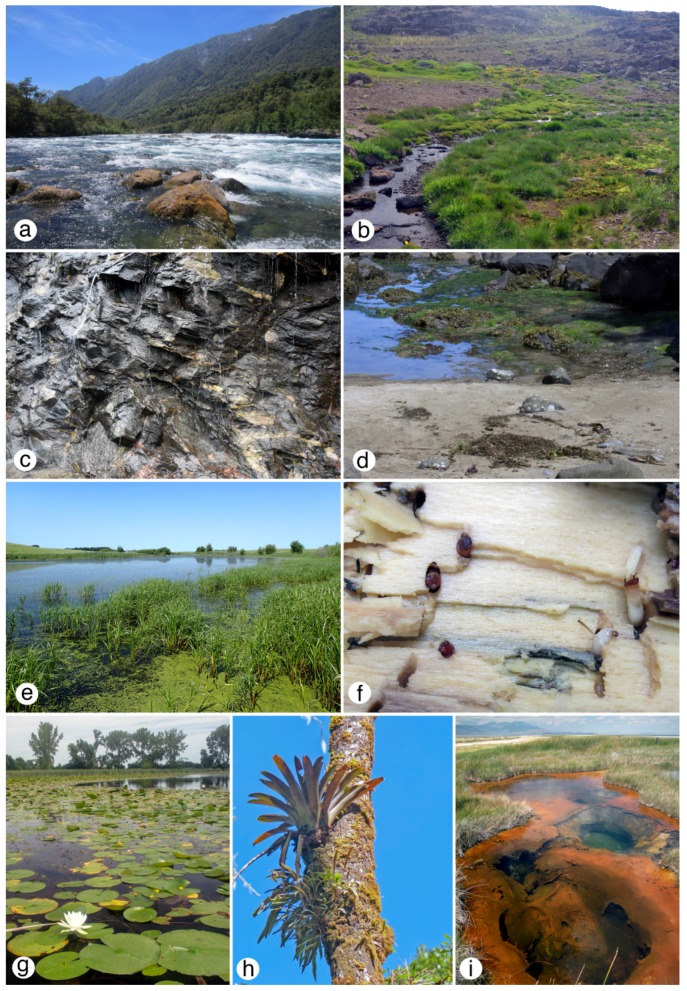
Diptera habitat: (**a**) Rio Petrohue, Chile; (**b**) Alpine headwater seepage, Oregon; (**c**) Bedrock seepage (madicolous zone), North Carolina; (**d**) Rocky intertidal and seaweeds, Oregon; (**e**) Prairie pothole marsh, Iowa; (**f**) Saturated wood (with *Axymyia furcata* larvae and pupae), Ohio; (**g**) Lake with macrophytes, Iowa; (**h**) Tank bromeliad (*Vriesia*), Costa Rica; (**i**). Hot spring, Oregon. All figures © G.W. Courtney.

**Figure 3:**
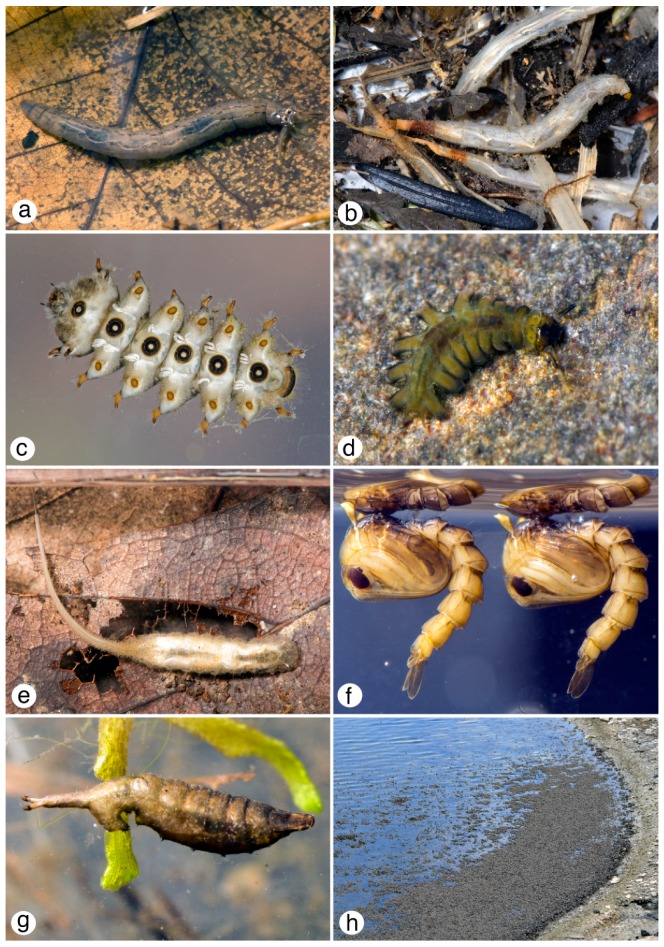
Diptera diversity: (**a**) *Tipula* (*Yamatotipula*) *tricolor* group (Tipulidae) larva, Iowa; (**b**) *Ptychoptera townesi* (Ptychopteridae) larvae, Oregon; (**c**) *Bibiocephala grandis* (Blephariceridae) larva, Oregon; (**d**) *Deuterophlebia vernalis* (Deuterophlebiidae) larva, Washington; (**e**) *Eristalis* (Syrphidae) larva, Iowa; (**f**) *Aedes aegypti* (Culicidae) pupae, laboratory colony; (**g**) *Setacera* (Ephydridae) puparium, Iowa; (**h**) *Ephydra* (Ephydridae) adults, Great Salt Lake, Utah. All figures © G.W. Courtney.

**Figure 4:**
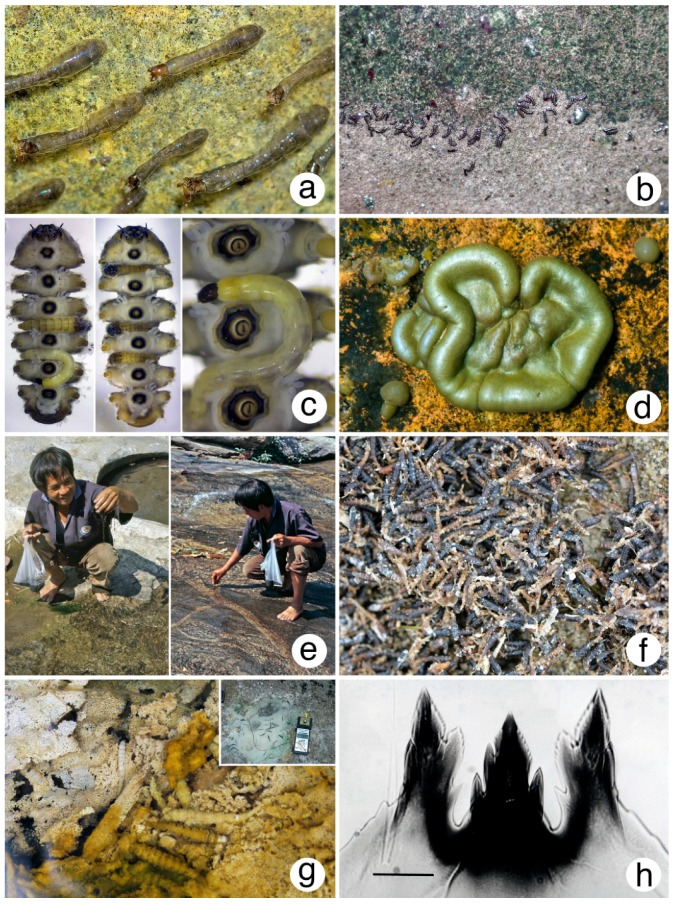
Diptera diversity & services: (**a**) *Simulium* (Simuliidae) larvae, Chile; (**b**) Line of grazing *Apistomyia* (Blephariceridae) larvae, Thailand; (**c**) *Neocurupira hudsoni* (Blephariceridae) larvae with symbiotic *Tonnoirocladius commensalis* (Chironomidae) larvae and pupae, New Zealand; (**d**) Cyanobacteria (*Nostoc parmeliodes*) containing *Cricotopus nostocicola* (Chironomidae) larva, Oregon; (**e**) Harvesting *Simulium* (Simuliidae) larvae for human consumption, Thailand; (**f**) Masses of *Ephydra* (Ephydridae) puparia, Great Salt Lake, Utah. (**g**) *Odontomyia* (Stratiomyidae) larvae in hot spring, Oregon (inset = larvae in hot spring, Yellowstone National Park); (**h**) *Parasimulium crosskeyi* (Simuliidae) larval hypostoma, Oregon. Figure 4a © S.A. Marshall; Figure 4b–h © G.W. Courtney, except inset of Figure 4g © E. Boyd.

All free-standing forms of water—permanent and temporary, flowing and stagnant, shallow and deep—have been exploited by flies (Figure 2). Only a small amount of water need be available to support dipteran development. The minute amounts of water held by leaf axils, bracts, and other plant cavities (phytotelmata) often support a specialized community of flies. Public health officials warned the public during the 2016 American outbreak of Zika virus fever that the offending mosquito vectors (*Aedes aegypti* and *A. albopictus*; Figure 3f) can develop in rainwater held by a discarded bottle cap [13]. Many aquatic Diptera, such as the Corethrellidae and more than 400 species of mosquitoes, develop in phytotelmata (Figure 2h) and other small, natural containers (e.g., hoofprints) that hold water [14,15,16]. Madicolous habitats—films of flowing water no deeper than 2 mm (Figure 2c and Figure 4b)—support a unique aquatic insect fauna, often composed primarily of Diptera, such as Thaumaleidae [17,18]. The water surface and splash and spray zones offer similarly thin water zones occupied by taxa such as the Dixidae and some Blephariceridae, Limoniidae, Psychodidae, and Stratiomyidae.

Plants provide multiple habitats for aquatic Diptera. For example, the immature stages of some chironomids, limoniids, syrphids, tanyderids, and tipulids, and all known axymiids inhabit saturated wood (Figure 2f), particularly along stream margins [19,20]. Some ephydrids mine the leaves and stems of aquatic plants [21], and limoniids, pediciids, and tipulids are frequent in leaf packs.

Some aquatic Diptera develop in subsurface habitats. Aquatic Diptera are typically absent from obligatory cave faunas, perhaps because of the visual limitations for the adults [22]. However, they can be found in interstitial habitats with water, such as the hyporheic and phreatic zones. Deeper habitats tend to be occupied by small, slender, flexible forms, such as the Chironomidae [23]. Likewise, Chironomidae, Ceratopogonidae, and various lower brachycerans (e.g., some Empididae, Pelecorhynchidae, and Tabanidae) are well represented in psammophilous habitats, which are characterized by fine sand either fully submerged or along the wetted margins of many water bodies. Aquatic dipterans that are highly specialized for subterranean existence typically lack pigment and eyes [24].

Few aquatic habitats are off limits for Diptera. Species of families such as the Chironomidae, Ephydridae, and Stratiomyidae, develop in thermal springs with temperatures often well above 35 °C (Figure 2i and Figure 4g). Stratiomyid larvae (*Odontomyia* nr. *occidentalis*) have been found in hot springs of Yellowstone National Park at torrid temperatures up to at least 73 °C [25] (Figure 4g), inset). On the other hand, larval chironomids have been taken from inside ice and frozen sediments [26], and G.W.C. has observed larval blepharicerids beneath anchor ice in rivers of the Canadian Rocky Mountains. A wide diversity of chironomids is found in acidic environments [27], and some species (e.g., *Chironomus acerbiphilus*) live in volcanic lakes with a pH as low as 2.0 [28].

More than 90% of aquatic Diptera inhabit freshwater. The remaining species are found in marine and brackish water habitats, primarily intertidal zones (Figure 2d) and estuarine marshes, but also saline pools and lakes. These species are members of at least 15 families: Canacidae, Ceratopogonidae, Chironomidae, Coelopidae, Culicidae, Dolichopodidae, Dryomyzidae, Ephydridae, Helcomyzidae, Heterocheilidae, Limoniidae, Muscidae, Psychodidae, Stratiomyidae, Tabanidae, and even Thaumaleidae, which can inhabit cliffside seepages that extend into the upper-intertidal zone and periodically are inundated with saltwater spray. Numerous species in several families (e.g., Coelopidae and Helcomyzidae) are associated with seaweed (Figure 2d), and their life cycles are coordinated with tidal rhythms [29]. Diptera are perhaps the only insects that have entered the open ocean beneath its surface, with the chironomid *Pontomyia* among the most unusual [30,31,32]. These and a few other chironomids, such as the epibionts on marine turtle carapaces, are truly submarine [33]; other chironomid species also probably live in the open ocean [34]. Alkali pools and salt lakes are marginal habitats for a limited set of life forms, providing enemy-free space to specialized aquatic Diptera, such as some Ephydridae [35].

Diptera are among the most abundant macro-organisms in aquatic environments. The highest levels of secondary production ever recorded are for black flies below a lake outlet in England: more than 1 million larvae per square meter [36]. Large rivers can support 600,000 larval black flies per square meter [37] and produce nearly a billion adults per kilometer per day [38]. A sewage plant in California can discharge an estimated 20 million psychodids daily [39]. Mosquito densities of 12.5 million adults per hectare [40] and nymphomyiid swarms so dense that the opposite side of the stream is obscured [41,42] also speak to extreme levels of productivity. Estimates of 40–50 billion chironomids produced per night from 200 hectares of waste-water stabilization ponds nearly bankrupt the mind [43]. Shore flies (Ephydridae) historically reached densities of more than 230 million flies per kilometer of shoreline along Great Salt Lake, Utah (Figure 3h), where vast windrows of their puparia (Figure 4f) piled more than 30 cm deep [44].

The entire panoply of trophic habits is well represented among aquatic Diptera [6]: collectors (e.g., Dixidae and Simuliidae), predators (e.g., Chaoboridae, Dolichopodidae, and Tabanidae), scrapers or grazers (e.g., Blephariceridae and Thaumaleidae), and shredders (e.g., some Psychodidae and Tipulidae). A few families (e.g., Chironomidae and Ephydridae) include all categories of feeding habits. Ingested food materials range in form and size from colloids and dissolved organic matter [45,46] to entire organisms, living or dead.

Diptera are structurally well adapted to life in water. Although larvae are legless, they often have cuticular modifications that allow purchase as they move through damp materials or adhere to substrates in flowing water. The larvae of Blephariceridae, Deuterophlebiidae, and Simuliidae are among the most specialized inhabitants of flowing waters, and all have structural features that permit locomotion on current-exposed substrates. Blepharicerid larvae, which often inhabit streams where current velocities exceed 2 m/s, show perhaps the greatest structural specialization. Their larvae have a compact body with six primary divisions, the first comprising the cephalic division, with a fused head, thorax and first abdominal segment (Figure 3c). This division and each of the remaining five have a highly specialized, ventral suctorial disc used to attach the larva to smooth rocks. Deuterophlebiid larvae, which often occur on the same rocks as larval blepharicerids, are dorsoventrally flattened and attach to rocks via elongate lateral prolegs that bear apical rows of crochets (Figure 3d). Larval simuliids use a crochet-tipped posterior proleg and a silk pad to adhere to submerged rocks and vegetation, where most species then extend modified labral fans into the current and strain food particles (Figure 4a).

The respiratory systems of aquatic Diptera are typically oligopneustic (e.g., metapneustic) or apneustic [47], and may be modified in various ways to accommodate life in aquatic habitats. For example, some groups, such as the Ptychopteridae and some Syrphidae, possess a slender, retractile terminal respiratory siphon that can be extended up to two or three times the body length (Figure 3b,e). Other remarkable respiratory modifications occur in certain culicids (e.g., *Coquillettidia* and *Mansonia*) whose larvae obtain oxygen by inserting a specialized siphon into submerged vegetation. Some larval chironomids can supplement their oxygen requirements in oxygen-poor habitats by using hemoglobin [47], which imparts the red body color and gives rise to the vernacular name “bloodworms”. Pupae of many aquatic species are adapted to life in both aquatic and terrestrial settings. The Simuliidae, for example, have pupal gills that can exchange gasses in and out of water, allowing for the chance that the pupa, fixed in its silken cocoon, is stranded when water levels drop [48]. Conversely, pupae of at least some Blephariceridae, which are glued to the substrate, desiccate if they are dry for a short period [49]. Eggs of most species of Diptera, even if deposited in terrestrial environments, have aquatic adaptations, such as an intrachorionic meshwork to facilitate gas exchange; otherwise, a single drop of rain can suffocate an egg.

## 4. Ecological Services

Diptera, given their diversity and abundance, drive ecosystem processes, both on land and in water. All species are of ecological importance, although the degree of their ecosystem influence varies in space and time and across taxa. In many cases, the ecological significance is simply not known, affording abundant opportunities for fundamental bionomic studies.

### 4.1. Ecosystem Engineers and Keystone Species

Although all species affect their environment, as least in the immediate vicinity of the individual, some species have a profound effect on the entire habitat. The effect is generally proportional to population size. Certain aquatic Diptera qualify as “ecosystem engineers”—those species that significantly alter their abiotic habitat and thereby affect the ecology of other organisms and related processes in the ecosystem [37]. The concept overlaps with that of “keystone species”—those organisms that have disproportionate effects on the function of ecosystems and the structure of communities [50].

Suspension-feeding larval Diptera help retain organic matter in lentic and lotic environments [37,51]. Small particles, colloids, and dissolved organic matter captured by suspension feeders, such as black flies, are packed into fecal pellets that are transported downstream. The repackaged organic matter then becomes available as food for microorganisms and macroinvertebrates, either during transport or after sedimentation. Mosquito larvae in standing water, such as natural pools and ponds, produce a rain of fecal pellets that similarly provide food for other organisms.

The daily transport of fecal pellets from larval simuliids past a line across some rivers can reach a stunning 429 tons of dry mass [52], roughly equivalent to 6000 elephants defecating (wet weight) into the river each day. These fecal pellets contain high amounts of carbon and nitrogen and provide substrate for bacteria and biofilms; thus, they contribute to the aquatic food web and fertilize riverbanks and floodplains [53]. Silk produced by larval black flies (Figure 4a) is sticky and gathers fine particles and colloids, helping to retain organic matter in the system, and the silk cocoons trap detritus and provide refuge for other macroinvertebrates [53]. Thus, silk of aquatic Diptera, such as simuliids, influences the development of periphyton and enhances the formation of tufa [54].

Organisms that by burrowing, eating, and defecating can turn and condition sediments are known as “bioturbators”. Through their activities, they can mobilize various compounds and nutrients, making them available to other organisms [55,56]. Bioturbators are well represented among aquatic Diptera. Chironomid larvae, which can reach densities of 150,000 per square meter, function much like earthworms, eating dead organic matter and defecating; these activities add significant aeration and nitrogen to the habitat, thereby transforming the plant and animal community [57].

Scrapers (grazers) can clean substrates of periphyton and other adherent debris, opening up colonization areas for other organisms. Densities of up to 1000 larval blepharicerids per square meter [58] can clean a significant amount of rocky surface area (Figure 4b).

Diptera that break down plant material, for example by shredding (e.g., *Tipula*, Figure 3a), provide a critical service by supplying both coarse and fine particulate organic matter that many organisms subsequently consume [59].

Predators can regulate populations and spatial distributions of other organisms and can structure aquatic communities. Predatory larvae of Chaoborus, for instance, maintain the structure of zooplankton communities in Canadian lakes [60].

### 4.2. Food Resources for Other Organisms

Aquatic Diptera, particularly the larvae, are a significant food resource for myriad invertebrates and vertebrates, and the fitness of many organisms is proportional to the supply. Even carnivorous plants can capitalize on aquatic Diptera emerging from in-stream pupae [61]. A large literature exists on the contribution of aquatic dipterans to the diets of vertebrates, such as birds, particularly those living in wetlands [62]. For instance, production of harlequin ducks is directly correlated with populations of black flies [63], and low availability of chironomids can reduce duckling survival of multiple species [64]. Aquatic Diptera, such as chironomids, are a significant part of the stream benthos and drift [65], and consequently are well represented in fish and invertebrate diets [66]. Even groups that seem relatively rare over an annual cycle (e.g., Blephariceridae) can be seasonally abundant and an important component of stream secondary production [67] and fish diets [68].

The dietary role of pupae of aquatic Diptera is less well studied than that of larvae. For eggs, it is virtually unaddressed. We can imagine, however, that if half a billion female black flies emerge from a kilometer of river, each with a clutch of about 120 eggs [38], the contribution of egg biomass and its ecological role must be significant. Similar calculations for other high-density aquatic Diptera, generally with high egg numbers per fly, also suggest a significant, although unknown, ecological contribution. Perhaps the eggs provide an important food resource for small arthropods and other invertebrates.

### 4.3. Symbioses

Each macro-organism is a community of symbiotic organisms functioning as a self-contained ecosystem. These symbiotes range in size from tiny unicellular forms (e.g., bacteria and protozoa) or smaller (i.e., viruses) to larger forms that approach the size of the host (e.g., nematodes and insects). Although considerable progress has been made in aquatic groups such as the Culicidae, exploration of symbiotic communities in aquatic Diptera is largely in its infancy.

Conspicuously underrepresented among aquatic Diptera are parasitoids. No members of the species-rich Tachinidae, for example, are considered aquatic. Some aquatic sciomyzids, however, are considered parasitoids of molluscs by some authors [69,70], and some predators of other aquatic Diptera approach parasitoid function. Larvae of the dryomyzid *Oedoparena glauca* feed inside the tissues of barnacles and, during low tides, search for new host barnacles [71]. Chironomid larvae are often found on various aquatic macroinvertebrates. In many cases, the relationship is merely one of opportunism or phoresy (e.g., *Tonoirocladius* on *Neocurupira* [72], Figure 4c), but in some cases, such as with the genus *Nanocladius*, the relationship is considered one of predator and prey [49] or parasitic [73]. Larvae of the chironomid Baeoctenus bicolor feed on the gills of unionid bivalve molluscs [74]. Some chironomid larvae take up residence in cocoons of other Diptera, such as the Simuliidae, and consume the pupal host, sometimes pupating in the host cocoon [48]. In yet other Chironomidae (e.g., certain species of *Cricotopus*), the larvae have a mutualistic relationship with Nostoc [75], a common cyanobacteria in many streams (Figure 4d).

Conversely, examples of aquatic Diptera infected with insectan parasitoids are spotty. Fully submerged taxa, such as the Simuliidae, are virtually free of insectan parasitoids, a benefit that extends to the terrestrial adults [48]. Among aquatic Diptera, the Ephydridae are most prone to harboring hymenopteran parasitoids, followed by Sciomyzidae, Stratiomyidae, and Tabanidae [76]. Hymenopteran parasitism is spotty or fortuitous in other groups [77].

Aquatic Diptera, however, are hosts of a vast diversity of other symbiotic organisms. Nearly 200 species of nonbacterial parasites and pathogens have been recorded in larval black flies [78]. More than 150 species of microsporidia alone are known from mosquitoes [79]. The bacterial community living on and in aquatic Diptera is extraordinarily diverse and differs among species and life stages and between genders [80]. A majority of the bacterial species are probably essential to the host. Thus, the ecological services of aquatic Diptera are actually delivered by a packaged ecosystem of diverse microorganisms that drive many of the host dipteran functions.

## 5. Societal Services

The range of services that aquatic Diptera can offer human society is limited only by the creativity of investigators. The following examples provide a glimpse of some of the service opportunities.

### 5.1. Bioassessment

Aquatic insects are excellent indicators of water quality [81,82]. As “canaries in the coal mine”, they often provide the first signs that an aquatic habitat is polluted or impaired. Among the Diptera, only the ubiquitous Chironomidae are routinely used in aquatic bioassessment. They are a standard part of the Environmental Protection Agency’s rapid bioassessment protocols [83]. Chironomids also are used as standard organisms in laboratory toxicity tests [84]. Certain taxa, however, might be particularly suited for detecting specific forms of pollution. Dixid larvae, which are uniquely adapted for life in surface films, are sensitive to surfactants and oils [85].

Improved detection of habitat impairment through bioassessment can be achieved with finer levels of identification [86]. The species level is ideal. Fine-scale identification, however, has conventionally been limited because many species of aquatic Diptera are still undescribed, cryptic species are frequent, and a large fraction of aquatic immatures remain unassociated with formally named adults [87]. Of the roughly 1225 Neotropical Ceratopogonidae, only 9% are known as larvae [88]. The situation is no better for some families even in well-studied areas. For instance, the larvae and pupae of perhaps only 10% of North American crane flies have been associated with their adults [4]. Limited use of aquatic Diptera in bioassessement, thus, probably stems in part from identification challenges. Other factors, however, are also at play, perhaps including ease of identification and taxon popularity. The Simuliidae, for example, are well known at the species level, with about 93% of the North American species known as pupae and about 98% as larvae [48]. Yet, they are rarely used in biotic assessment, even though metrics are available [89].

Diptera uniquely possess giant (polytene) chromosomes in many of their tissues, particularly the salivary glands. The use of polytene banding patterns has allowed increased identification capability. The technique, however, is sufficiently developed only in the Chironomidae, Culicidae, and Simuliidae, and even in these groups the taxonomic coverage is spotty [90,91,92]. The technique also remains arcane to most users, typically requiring a level of training beyond that for morphological identification. Consequently, the utility of polytene chromosomes in water-quality assessment has not been exploited. Nonetheless, much as structural deformities of chironomid larvae can be used to detect heavy-metal toxicity [93], the polytenes also can indicate environmental insults that might not be revealed at an organismal level. Stress puffs and other aberrations in the complement can indicate environmental (e.g., chemical or thermal) stresses [94,95].

The advent of molecular techniques and the continuing refinement and power of molecular methodology, accompanied by cost reductions and general availability of the procedure, offer the opportunity for all aquatic species to contribute information about water quality [96,97]. Time will be required, however, to populate the molecular databases so that sequences of field-collected specimens can be compared with those of known reference species.

Aquatic Diptera also can serve as indicators of specialized or threatened communities and habitats. Some black flies in the genus Parasimulium are associated with ancient forests, and could be sensitive indicators of forest health [98]. The Ephydridae and Sciomyzidae, because of their abundance and diversity, have been considered among the most important dipterans in freshwater wetlands of North America [62].

### 5.2. Paleoecology and Climate Change

The importance of fossil Diptera as indicators of palaeoclimates is well established. Foremost in this role are the Chironomidae [99,100,101], which are diverse, widespread, and have larvae that are abundant in aquatic habitats and provide fossil remains that can be readily identified. The fossil remains include mostly larval head capsules and mouthparts (e.g., mandibles and hypostoma (postmentum)), which are well-sclerotized in many aquatic flies (e.g., Simuliidae, Figure 4h) and can persist in lacustrine deposits for thousands of years.

Because these structures permit identification to family, genus, and sometimes species, and we often have detailed knowledge of the abiotic and biotic requirements of these taxa, they can be used to establish temperature proxies [102] or infer past (and future) environmental changes [103,104,105]. Dipteran bioindicators might be particularly sensitive at higher latitudes [106,107] or along elevational gradients [105,108]. In addition to the use of well-sclerotized body parts of aquatic Diptera, molecular analyses of population genetic structure also can reveal a signature of past environmental disturbances, such as glaciation. Species that are geographically restricted and habitat-specific at high elevations have limited options to respond and are, therefore, particularly vulnerable to climate change and atmospheric deposition; consequently, they can be sensitive indicators of climate change and other environmental disturbances [109].

Although chironomids have been the focal taxon for paleoecological investigations, presumably because of their abundance in the fossil record, other groups, such as the Simuliidae [110], Chaoboridae [106], Ceratopogonidae [111], and even Sciaridae [112], have also provided insights into past environmental changes. These groups also can be used as historical indicators of changes in community structure. For example, because the presence of certain species of *Chaoborus* is determined in large part by the presence or absence of fish, the larvae have been used as paleoindicators of fish populations [113].

### 5.3. Biological Control

The use of aquatic Diptera as biological control agents has typically taken the form of predators against blood-feeding pests and vectors and of herbivores against aquatic weeds. The 90 or so species of the genus *Toxorhynchites* are large mosquitoes that, as adults, cannot cut tissue to take blood and, as larvae, prey on other mosquitoes. They have been regarded as biological control agents of pest and vector mosquitoes for more than 100 years and have been used in this context with varying levels of success [114]. Although not specifically used for biological control, the larvae of seven families of aquatic Diptera have been documented as predators of black flies [115].

Larval Sciomyzidae are obligate predators (or parasitoids) of snails that serve as intermediate hosts of trematodes causing schistosomiasis (bilharzia) and fascioliasis of humans and livestock. Their potential as biological control agents is, thus, high and has seen some field success; greater reliance on sciomyzids is anticipated as resistance to antihelminthics and concerns about chemical applications in wetlands increases [69,70].

Herbivorous aquatic Diptera offer potential as biological control agents of noxious aquatic plants. Specialized algal feeders among the Ephydridae [21] could be used as biological control agents if the algae were to experience explosive growth. Plant-mining ephydrids of the genus *Hydrellia* have been used successfully as biological control agents against wetland hydrilla weeds [116,117]. Other herbivorous Diptera (e.g., Chironomidae) might offer potential in biological control. However, some chironomids are pests of agricultural crops, such as rice [118], suggesting that the degree of plant specificity for certain species is too weak to warrant consideration.

### 5.4. Bioproducts

In a widely publicized study, the economic value of insects in the United States was selectively assessed, beginning with an estimate of $60 billion spent annually on fishing (sport and commercial), hunting, and bird watching, and then reasoning that the essence of these activities traces in good part to insects as dietary items for wildlife [119]. Teasing apart the contribution by aquatic, versus terrestrial, insects has not been attempted, but we can infer that it would be significant. Aquatic Diptera, for example, are an integral part of many aquaculture programs as dietary components for fish [120].

Insects have been promoted as an abundant and nutritious (e.g., high-protein) source of human food for at least 3000 years [121]. The role of insects in human diets has typically been skewed toward terrestrial groups. Aquatic Diptera, nonetheless, have historically figured in the diets of some peoples. Native Americans (e.g., certain bands of Paiutes) gathered “bushels” of puparia of *Ephydra hians* (koo-tsabe) along the shores of Mono Lake, California, and to a large degree subsisted on them at certain times of year, at least until the tribes were relegated to reservations [44]. Four families of aquatic Diptera—Chironomidae, Culicidae, Simuliidae, and Tipulidae—have been identified as having the greatest potential as human food in today’s world, based in part on the possibility for bulk harvesting [121]. Localized use of aquatic Diptera for human food has been observed when circumstances provide high numbers of larvae that are easily harvested [122,123]. We have observed locals in Thailand gathering larvae of the large black fly *Simulium rudnicki* from bedrock outcroppings (Figure 4e) and mixing them with lemon juice and chili peppers for raw consumption.

Pharmaceutical possibilities derived from the chemistries of aquatic Diptera are little explored. The reason might relate to the dull colors of aquatic animals generally, a trend noted well over 200 years ago and later pondered for aquatic insects, by the renowned ecologist G. Evelyn Hutchinson [124]. Bright colors are often associated with defensive chemicals, typically derived from secondary plant compounds; subdued coloration, thus, might suggest less reliance on chemicals for defense. However, as the lesson from drab aquatic dytiscid beetles has shown [125], much potential exists for the cornucopia of chemistries of aquatic insects. We suggest that chemical prospecting among aquatic Diptera would be worthwhile for discovering novel compounds with pharmaceutical and other useful properties.

### 5.5. Bioinspiration and Biotechnology

The aquatic adaptations of Diptera provide a wellspring of solutions to modern engineering challenges. These adaptations are the result of millions of years of evolutionary experiments in natural environments, a difficult result to achieve in a short time in laboratories that often rely on extreme heat and pressure to create comparable products, typically with undesirable production of hazardous wastes and non-recyclable materials. Tapping the wealth of bioinspiration requires a good knowledge of dipteran taxonomy and natural history, coupled with an understanding of chemistry, materials science, mechanics, and physics. Diverse disciplinary combinations and interests are seldom found in one intellectual package, but they can be realized through interdisciplinary collaborations that work to achieve fundamental understanding of aquatic dipteran adaptations as a means of solving engineering problems in human society. Aquatic Diptera are little explored from an engineering or biotechnological perspective, so a profitable frontier awaits the curious and the enterprising.

The field of robotics has often capitalized on an arthropod-leg model. Yet, the possibility of dipteran larval locomotion in aquatic environments, which often involves mechanisms for burrowing [126] and swimming [127], has garnered much less attention. Burrowing robots based on aquatic larval dipteran models might find application, for example, in below-surface planetary exploration and medical procedures.

Controlled adhesion has been a popular subject among bioengineers, particularly using geckos and a few terrestrial insects. The six ventral suctorial discs of blepharicerid larvae are true hydraulic suckers (Figure 3c), and a description of their function has been analyzed in real time [128]. A mechanical analysis that focuses on their materials properties and the physics of their adherence–release could provide insights into novel artificial means of locomotion in fluids.

Investigations of biotechnological applications for silk produced by aquatic insects have lagged far behind those for terrestrial organisms, such as spiders and silkworms. These studies are even more poorly represented by aquatic Diptera, despite a remarkably diverse library of silk types among species, even within a family, and their functional properties, such as immediate adhesion in water. Some progress has been achieved in understanding the structural properties and genomics of silk proteins [129,130]. Silk, which is a composite biopolymer (i.e., protein fibers bonded by a matrix), differs among species in its degree of adhesion and resistance to microbial breakdown in aquatic environments [131].

Extremophilic aquatic Diptera offer enormous biotechnological potential, particularly in harsh terrestrial and extraterrestrial environments. Tolerance of abiotic extremes is represented within single families, such as the Chironomidae, and include tolerance of low and high temperatures, oxygen deprivation, high salinity, low pH, and desiccation [132]. Thermal tolerance by enzymes, tissues, and other materials operates in aquatic Diptera, such as the Stratiomyidae, that inhabit hot springs (Figure 2i and Figure 4g). The larval cuticle of stratiomyid larvae is impregnated with hexagonal calcium carbonate crystals, providing a tough barrier that perhaps also aids adjustment to life in low-pH environments of hot springs. Similarly informative at the lower temperature extremes are aquatic dipteran larvae, notably of chironomids (e.g., *Diamesa* spp.), that survive subfreezing temperatures for extended periods. Some of these cases involve freeze tolerance, accompanied by dehydration and supercooling down to −14 °C [133,134]. The gut bacteria of the petroleum fly, *Hylaeomyia petrolei*, are solvent-tolerant and their enzymes retain functionality in organic solvents, suggesting industrial and bioremediation utility [135]. Larvae of the African sleeping chironomid *Polypedilum vanderplanki* exhibit extreme desiccation tolerance (anhydrobiosis), with various protection mechanisms that permit revival up to 17 years later—extraordinary, particularly for relatively large organisms (7–8 mm long) [136]. These larvae experience severe DNA damage but have an efficient repair mechanism [137]. Chironomids generally have small genome size, including the tiniest genome known for an insect (*Clunio tsushimensis*), and sometimes the small size is more so for extremophiles [138], suggesting that gene investigations could be rewarding for understanding adaptation to extreme environments.

### 5.6. Forensic Sciences

Aquatic Diptera that function as decomposers akin to terrestrial muscoid flies, such as the Calliphoridae, are under-represented in aquatic habitats, particularly when a corpse is entirely submerged. Carcasses along shorelines and floating on the water’s surface offer an aquatic–terrestrial interface that is likely to include dipteran representatives of both environments. Some shore-inhabiting ephydrids consume small animal carcasses on beaches [21], suggesting forensic potential if they also use larger corpses. Forensic cases involving a dead human in water commonly feature the presence of chironomids; their diversity, abundance, ubiquity, and species-specific characteristics make the Chironomidae potentially useful in forensic investigations [139]. *Ad hoc* examples of the use of aquatic Diptera in forensic investigations have been documented. Pupal exuviae of the black fly *Prosimulium fuscum* found on a submerged car helped convict a murderer by countering his purported timeline of events [139].

## 6. Service Tradeoffs

Aquatic Diptera present a paradox. They can provide vital services to ecosystems and humans, but the same species also can cause health, economic, and ecological problems when their activities intersect or interfere with human interests or when the species become invasive.

Dipteran silk has a remarkable ability for sorbing pesticides from water. Consequently, it offers both benefits and risks. The silk provides a potential means of water purification but creates an undesirable mechanism for instant access of pesticides into the food web [140]. Similarly, although aquatic Diptera can serve as bioindicators of heavy metals and as fish food in aquaculture programs, they also can acquire and biomagnify metals, either naturally from their habitat or from pollution, and transfer the compounds into the food web [141,142]. Burrowing larvae can increase the flux of organismally desirable materials and render them available to the aquatic community, but they also can dredge up toxicants that are subsequently ingested by other organisms [56].

Although some herbivorous ephydrids (*Hydrellia* spp.) have been used as biological control agents against wetland weeds [116,117], some species can damage water-dependent and irrigated crops such as rice and watercress [143], requiring a certain degree of caution in using at least some species of *Hydrellia*. The brine fly *Ephydra hians* (koo-tsabe), which historically was a valued source of human food, is now considered a contaminant of cyanobacteria (“Spirulina”) used as a human and animal food supplement [144].

Invasive aquatic insects that play a well-integrated ecological role in their native system can have calamitous effects in their new ecosystem [145]. Mosquitoes provide particularly prominent examples, both as vectors of disease agents and as competitors of native taxa [146]. An invasive chironomid on Signy Island 600 km off the coast of Antarctica is transforming peat bogs by increasing their depth and contributing massive amounts of nitrogen; if it reaches Antarctica, it is predicted to have devastating consequences [57].

Tradeoffs are often driven by the stark environmental differences between the immatures and the adults. Classic examples involve a beneficial aquatic stage and a terrestrial pest or vector stage. Habitat eutrophication, typically caused by human influences, promotes population explosions of aquatic flies, particularly psychodids and chironomids, which specialize in these habitats [147]. Psychodid larvae (e.g., *Tinearia alternata*) can clean filters in water-treatment facilities by grazing accumulated films that otherwise block the interstices [148]. On the other hand, adults of the same species can cause asthmatic attacks in workers at sewage plants [149]. Chironomid larvae, by virtue of their abundance and rapid life cycles, can be beneficial as bioindicators, food for other organisms, and recyclers of organic matter. Nevertheless, numerous species that emerge en masse from eutrophic waters cause problems by entering facial orifices; asphyxiating cattle; soiling automobiles and paint jobs; clogging air conditioners and water-treatment equipment and channels; rendering roads and airport runways slippery; and causing allergic reactions such as asthma and rhinitis in humans [118,147].

The immature stages of blood-feeding flies, especially the Ceratopogonidae, Culicidae, Simuliidae, and Tabanidae, are important components of the aquatic food web, serving as prey for many organisms, conditioning substrates, and recycling nutrients. However, the adults of the same species cause nuisance problems for humans, domesticated animals, and wildlife, and transmit disease organisms. Blood feeding discourages outdoor activities and tourism, depresses economies, and influences wildlife behaviors such as migration and nesting [48].

## 7. Sustainability or Extinction

Protecting ecological function and exploiting the service potential of aquatic Diptera for humans requires sustainable populations. This objective is often counter to the need to control fly populations, such as those that are involved in transmitting disease agents. Extracting the beneficial attributes of aquatic Diptera that also can be pests or vectors depends on wise and adaptive management that views the system holistically [150].

Abundance, or even superabundance, does not guarantee perpetuity of a species. Some of the most abundant species on Earth have become extinct. The passenger pigeon and Rocky Mountain locust are well known examples [151]. The formerly superabundant black fly *Simulium vampirum*—once estimated at 7 billion pupae across a rocky weir in Canada [152]—and *S. colombaschense*—once so abundant that the blood-sucking female flies killed nearly 22,000 domesticated animals along the Danube River [153]—are far less numerous today, even scarce, as a result of control and habitat alteration [48,154].

A number of aquatic Diptera are threatened, endangered, or extinct, although the full picture is woefully inadequate given the state of knowledge for most species. Aquatic Diptera that are rarely encountered do not necessarily imply some level of vulnerability, but rather unknown or under-collected habitats [20]. In the well-known fauna of the United Kingdom, a mosquito and two ceratopogonids are considered “near threatened” [155]. The IUCN Red List highlights one blepharicerid and one tabanid as vulnerable, one blepharicerid and one psychodid as endangered, and one tabanid as extinct [156]. Perhaps one species of simuliid has become extinct as a result of habitat alteration [154]. Two genetically distinct simuliid forms were driven to extinction by vector control in onchocerciasis programs before their status as species could be evaluated [157,158].

## 8. Conclusions

Any aquatic habitat, large or small, is likely to support a population of one or more species of flies. At least 41 families and about 46,000 species of Diptera are associated with a liquid medium in one or more life stages. Aquatic flies serve a wide variety of environmental roles, from dietary items to habitat modifiers. Their services have been co-opted for human benefit as bioindicators of historical and current environmental conditions, biological control agents, and forensic indicators. They also provide a largely untapped source of bioproducts and inspirational models for engineering applications.

Although flies are typically the most abundant members of the freshwater insect community, the vast majority of species are poorly known. Many species remain to be discovered, the majority have not been associated with their terrestrial adult stages, and virtually nothing is known about the bionomics of most species other than, at best, anecdotal information or data on a collection label. Opportunities, therefore, abound not only for taxonomic and natural history investigations, but also for collaborations between the people who study aquatic flies and chemists, engineers, physicists, materials scientists, and other investigators. Aquatic flies, over evolutionary time, have solved some of the greatest environmental challenges. Understanding the role of aquatic flies in their ecosystems, and capitalizing on their adaptive solutions to aquatic life for the benefit of society, are worthy objectives.

## Figures and Tables

**Table 1:** Diversity and richness of aquatic Diptera.

Family or Superfamily	Total Species ^1^	Aquatic Species ^2^	Predominant Trophic Group; Habitat
Ceratopogonidae	5902	5182	collectors and predators; diverse lentic and lotic
Chaoboridae	89	89	predators; lentic
Chironomidae	7290	7090	all trophic groups; all aquatic habitats
Corethrellidae	111	111	predators; lentic (phytotelmata) and lotic (hyporheic)
Culicidae	3725	3725	collectors and some predators; lentic
Dixidae	197	197	collectors; lentic and lotic surfaces
Simuliidae	2335	2335	collectors; lotic
Thaumaleidae	183	183	scrapers; madicolous
Ptychopteridae	80	80	collectors; springs (mud)
Tanyderidae	40	40	collectors; lotic (gravels, saturated wood)
Blephariceridae	330	330	scrapers; lotic (rocks)
Deuterophebiidae	14	14	scrapers; lotic (rocks)
Nymphomyiidae	8	8	collectors/scrapers; lotic (mosses)
Axymyiidae	9	9	shredders; lotic margins (saturated wood)
Bibionidae	1102	1	collectors?
Scatopsidae	407	5	collectors; tree holes
Psychodidae	3026	1988	collectors and scrapers; lentic and lotic
Tipuloidea	15,803	11,062	all trophic groups; lentic and lotic
Cylindrotomidae	71	—	—
Limoniidae	10,813	—	—
Pediciidae	506	—	—
Tipulidae	4413	—	—
Stratiomyidae	2690	928	collectors; lentic, madicolous, thermal springs
Athericidae	133	133	predators; lotic
Oreoleptidae	1	1	predators; lotic
Pelecorhynchidae	49	49	predators; streams and swamps
Tabanidae	4434	4434	predators; lentic and lotic
Dolichopodidae	7358	3182	predators; lentic and lotic
Empididae	3142	671	predators; lotic
Lonchopteridae	65	2	collectors; freshwater shores
Phoridae	4202	17	collectors; lentic
Syrphidae	6107	1341	collectors; lentic and lotic margins (saturated wood)
Coelopidae	35	35	collectors and shredders; marine intertidal (seaweed)
Dryomyzidae	30	3	predators; marine intertidal
Helcomyzidae	12	12	collectors and shredders; marine intertidal (seaweed)
Heterocheilidae	2	2	collectors and shredders; marine intertidal (seaweed)
Sciomyzidae	618	194	predators; wetlands
Canacidae	323	323	scrapers; marine intertidal (seaweed) and lotic
Ephydridae	1994	1251	collectors and shredders; lentic, lotic (margins) and marine intertidal
Muscidae	5218	701	predators; lentic and lotic
Scathophagidae	419	150	shredders and predators; lentic
Sarcophagidae	3094	87	collectors and shredders; lentic (incl. phytotelmata)
Totals	80,549	45,965	

^1^ Ordering of families and species numbers are from [8]. ^2^ Species numbers are from [9], except those for Simuliidae [10], Bibionidae (aquatic number from [11]), Scathophagidae and Sarcophagidae (calculated from North American percentages [6]), and all numbers for Tipuloidea [12]; an estimated 200 species of Chironomidae and 720 species of Ceratopogonidae are terrestrial.
